# Network based analysis identifies *TP53*m-*BRCA1/2*wt-homologous recombination proficient (HRP) population with enhanced susceptibility to Vigil immunotherapy

**DOI:** 10.1038/s41417-021-00400-x

**Published:** 2021-11-16

**Authors:** Elyssa Sliheet, Molly Robinson, Susan Morand, Khalil Choucair, David Willoughby, Laura Stanbery, Phylicia Aaron, Ernest Bognar, John Nemunaitis

**Affiliations:** 1 Southern Methodist University, Department of Mathematics, Dallas, TX USA; 2 University of Toledo, Department of Medicine, Toledo, OH USA; 3 University of Kansas School of Medicine, Wichita, KS USA; 4 Ocean Ridge Biosciences, Deerfield, FL USA; 5 Gradalis, Inc, Carrollton, TX USA

**Keywords:** Ovarian cancer, Biomarkers

## Abstract

Thus far immunotherapy has had limited impact on ovarian cancer. Vigil (a novel DNA-based multifunctional immune-therapeutic) has shown clinical benefit to prolong relapse-free survival (RFS) and overall survival (OS) in the *BRCA* wild type and HRP populations. We further analyzed molecular signals related to sensitivity of Vigil treatment. Tissue from patients enrolled in the randomized double-blind trial of Vigil vs. placebo as maintenance in frontline management of advanced resectable ovarian cancer underwent DNA polymorphism analysis. Data was generated from a 981 gene panel to determine the tumor mutation burden and classify variants using Ingenuity Variant Analysis software (Qiagen) or NIH ClinVar. Only variants classified as pathogenic or likely pathogenic were included. STRING application (version 1.5.1) was used to create a protein-protein interaction network. Topological distance and probability of co-mutation were used to calculated the C-score and cumulative C-score (cumC-score). Kaplan–Meier analysis was used to determine the relationship between gene pairs with a high cumC-score and clinical parameters. Improved relapse free survival in Vigil treated patients was found for the *TP53*m-*BRCA*wt-HRP group compared to placebo (21.1 months versus 5.6 months *p* = 0.0013). Analysis of tumor mutation burden did not reveal statistical benefit in patients receiving Vigil versus placebo. Results suggest a subset of ovarian cancer patients with enhanced susceptibility to Vigil immunotherapy. The hypothesis-generating data presented invites a validation study of Vigil in target identified populations, and supports clinical consideration of STRING-generated network application to biomarker characterization with other cancer patients targeted with Vigil.

## Introduction

Ovarian cancer is the third most common gynecologic cancer, and it carries the worst prognosis and highest mortality rate of gynecologic cancers [1–3]. Mortality from ovarian cancer is three times that of breast cancer [3, 4]. Genetically, the majority of ovarian cancer cases are *BRCA* wild type (*BRCA*wt) but more than one-fifth of cases are attributable to mutations in tumor suppressor genes, with 65–85% of the mutations being in germline *BRCA* genes (g*BRCA*) [5, 6].

Standard treatment of resectable newly diagnosed stage III/IV ovarian cancer involves surgical resection and adjuvant or neoadjuvant chemotherapy [7, 8]. Unfortunately, nearly 75% of this patient population who undergo standard treatment will experience recurrence following frontline therapy [7, 9]. The establishment of more effective therapies for ovarian cancer is essential.

Major advancements in ovarian cancer maintenance therapy came with the advent of poly(ADP-ribose) polymerase (PARP) inhibitors (PARPi) [10], which are currently approved in newly diagnosed and recurrent ovarian cancer maintenance. PARP inhibitors target the PARP family of proteins (PARP1, PARP2, PARP3), which are a central component of the DNA repair machinery [11, 12]. When administered to patients with intrinsically deficient DNA repair including mutations in *BRCA* or other homologous recombination genes, PARPi results in synthetic lethality [12–14]. Benefit of maintenance PARPi however is limited in newly diagnosed patients who are *BRCA*wt or homologous recombination proficient (HRP) molecular profile [15, 16].

Vigil is an autologous tumor DNA immunotherapy transfected with a plasmid encoding granulocyte-macrophage colony-stimulating factor (GM-CSF) and bifunctional short hairpin RNA inhibitor against furin. Furin is an enzyme essential for cleaving TGF-beta into its active form [17]. Vigil was designed to enhance the immune system’s potency against cancer in three ways: first, Vigil introduces the individual tumor neoantigen repertoire to the immune system. Second, Vigil enhances differentiation and activation of immune cells via GM-CSF, a cytokine important to immune activation at both the peripheral and marrow levels. Finally, Vigil inhibits cancer expressive TGF-beta, thereby decreasing immunosuppressive activity of TGF-beta. Functional immune activation of Vigil in correlation with clinical benefit has been demonstrated via ELISPOT assay [18, 19]. Moreover, Vigil appears to increase CD3+/CD8+ T cell circulation in advanced solid tumor patients and expands MHC-II expression activity, as determined by NanoString analysis, in correlation with clinical benefit [20, 21]. Safety and evidence of efficacy of Vigil has been evaluated in several tumor types in addition to ovarian cancer [18, 19, 22–26].

A randomized double-blind placebo-controlled study (VITAL trial) of Vigil versus placebo as maintenance therapy for frontline stage III/IV ovarian cancer recently demonstrated clinical benefit in terms of recurrence-free survival (RFS) from randomization (done just prior to maintenance therapy initiation) (HR = 0.51; CI 90% 0.30–0.88; *p* = 0.02) and overall survival (OS) (HR = 0.49; 90% CI 0.24–1.0 *p* = 0.049) in patients with *BRCA*wt tumors [17]. Additional post-hoc analysis demonstrated further clinical benefit in RFS and OS (HR 0.386; 90% CI 0.199–0.750; *p* = 0.007 and HR 0.342; 90% CI 0.141–0.832; *p* = 0.019) from randomization in patients with HRP molecular profile [27]. We hypothesize that intact DNA repair mechanisms of *BRCA*wt, HRP ovarian cancer may be important for Vigil efficacy, possibly related to higher degree of clonal versus subclonal neoantigens available for anticancer immune stimulation [27, 28]. We now describe further molecular analysis in coordination with clinical benefit parameters of genomic variant data in all patients involved in the VITAL trial. We seek to identify significant genomic variants, meaningful variant combinations, and relevant genes at the intersection or “hub” of ovarian cancer pathways which provide proof of principle to a novel clinically applicable method of biomarker assessment.

## Methods

### Data management and design

Tumor annotated DNA polymorphism data was generated by Ocean Ridge Biosciences (ORB) (Deerfield Beach, Florida), across 981 validated genes for all patients who entered into the Phase IIb double-blind randomized placebo-controlled trial (NCT02346747) comparing Vigil and placebo in Stage III/IV resectable ovarian cancer. Patient demographics, trial design, and vaccine manufacturing were previously described [17]. Patients were enrolled following IRB approved written consent. DNA samples of malignant tissue were analyzed from all 91 patients entered into trial and results were compared to clinical endpoints prospectively identified in the study statistical plan. Gene variants were classified by either Ingenuity Variant Analysis software (Qiagen, Valencia, CA) or the NIH ClinVar database (current versions as of 10 February 2020) [29].

Only gene variants which were determined to be pathogenic or likely pathogenic were included and were referred to as pathogenic mutations. Individual gene sets of pathogenically mutated genes for each patient in the trial were generated. An overall gene set was then constructed by taking the unique union (i.e., combining all of the individual patient gene sets and removing duplicated genes) of the individual gene sets. A binary mutation matrix was constructed from this overall gene set such that element (*i, j*) of the mutation matrix was equal to 1 if patient *i* had a pathogenic mutation in gene *j* and equal to 0 if patient *i* was wild type in gene *j*. Visual display of this mutation matrix of all patients is shown in Supplementary Fig. 1. The mutation matrix allows for visualization of common mutations and genetic profiles but does not explore the functional relationship between genes.

### Tumor mutation burden analysis

Annotated DNA polymorphism data generated from sequencing the coding regions of 981 tumor-related genes of the Roche panel from both PBMC (germline) and tumor cells (somatic) was utilized to determine the tumor mutational burden (TMB) for each patient [30]. Common DNA polymorphisms, as evidenced by their presence in dbSNP database v. 151 or the 1000 genome project database (Phase III version 5b), were removed from consideration as were any variants that were not classified as exonic. Somatic polymorphisms were defined as SNPs or insertions/deletions that were present at a 5% frequency or greater in the tumor DNA of a patient while being absent from the germline sample from the same patient. TMB was calculated as the sum of the synonymous and non-synonymous somatic mutations across the coding regions of the 981 genes divided by the length in Mb of the consensus coding region sequence (CCDS) of the sequenced exonic regions of these genes (2,702,326 bp). Patients with TMB scores ≥10 were classified as high TMB, and <10 were classified as low TMB.

### STRING and topological distance

The STRING database has been maintained since 2000 by the Swiss Institute of Bioinformatics, CPR - Novo Nordisk Foundation Center Protein Research, and EMBL - European Molecular Biology Laboratory [http://string-db.org/] [31–39]. Pathogenically mutated genes were inputted into the STRING (Search Tool for the Retrieval of Interacting Genes/Proteins) application (Version 1.5.1) in Cytoscape (Version 3.8.0) to gauge functional interaction. The STRING application generates a network for the input genes which consists of genes and their interactions, represented by nodes and the lines which connect them, referred to as vertices and edges, respectively. Genes are only connected via edges in the network if there is evidence they interact from published literature and high throughput experimental data. STRING uses this information to assign confidence scores, which are denoted as *s*(*i*, *j*), to each interaction or edge. Individual STRING scores are produced for each of the interaction types and these scores are integrated to give a combined confidence score, *s*(*i*, *j*), between each pair of proteins. Each protein-protein interaction (PPI) score is bound between 0 and 1 which indicates how likely STRING judges the particular interaction to be true, given available evidence. Next, edge weights (*w*) between each pair of genes are calculated according to the following formula: *w*(*i, j*) = 10(1−*s*(*i*, *j*)).

The score, *s*(*i*, *j*) was subtracted from one so that intuitively a small weight corresponds to strong evidence of a biological interaction between a gene pair and multiplied by 10 to shift the values to the desired scale [40]. Dijkstra’s Algorithm was then used to calculate the length of the shortest weighted path between genes denoted, *d*(*i*, *j*) by summing over the weighted edges that connect them and systematically finding the shortest weighted path. Genes with distance ≤3.8 was defined as the bottom quarter. Intuitively, when a gene pair has a low topological distance, *d*(*i*, *j*), the genes may interact biologically.

### C-scores

The independent concepts of patient mutation profiles and the STRING Network are integrated by C-score. The probability of co-mutation for every pair of genes in the overall gene set was calculated. The probability (*P*) of a co-mutation was defined as the total number of the 91 patients who have a mutation in both of the genes in the given gene pair divided by a measure of the total number of times both genes are mutated individually, given by:

$$P\left( {i,j} \right) = \frac{{\left| {G\left( i \right)\mathop { \cap }\nolimits G\left( j \right)} \right|}}{{\sqrt {m\left( i \right) \ast m\left( j \right)} }}$$

[41]. Where $|G(i)\mathop { \cap }\nolimits G(j)|$ represents the number of individual tumors where both genes *i* and *j* are mutated, and *m*(*i*) and *m*(*j*) are the cumulative mutations of genes *i* and *j*, respectively. The range of *P*(*i, j*) is between 0 and 1 where *P*(*i, j*) = 0 indicates that genes *i* and *j* never co-mutate and *P*(*i, j*) = 1 means the genes will always co-mutate.

The probability of co-mutation and the topological distance between genes in the STRING network were then combined to calculate a C-score, denoted *C*(*i, j*), to quantify the likelihood that the genes interact functionally, termed “putative genetic interactions” [41]. The C-score is calculated by dividing the probability of co-mutation by the topological distance from the STRING network squared.

$$C\left( {i,j} \right) = \frac{{P\left( {i,j} \right)}}{{d(i,j)^2}} = \frac{{|G(i)\mathop { \cap }\nolimits G(j)|}}{{\sqrt {m(i) \ast m(j)} d(i,j)^2}}$$

Further, the cumulative C-score for a gene *i* is denoted, $cumC(i) = \mathop {\sum}\nolimits_{i \ne j} {C(i,j)}$ [41]. A gene with a high cumulative C-score is more likely to co-mutate with genes close to it in the STRING Network. In order to determine the significance for these C-scores, a permutation test is performed. We began by reshuffling the mutation profile of each patient through preserving the number of mutations of each patient and randomly assigning new mutations. Then we followed the above methodology to calculate “simulated C-scores”, *C*_*s*_(*i, j*). The *p*-value was then calculated by taking the total number of times the simulated C-score was greater than or equal to the actual C-score and dividing by the number of trials performed (*n* = 10,000) [41].

$$p = \frac{{|C_s\left( {i,j} \right) \ge C\left( {i,j} \right)|}}{{10,000}}$$

### Pathway analysis

A list of pathways associated with each gene was extracted and binned into seven color coded categories that included DNA repair, chromosomal organization and transcription, regulation of translational and post-translational modification, immunity, other pathways, other cancer genes, and undefined.

### Survival analysis

RFS and OS relationships of patients with varying mutational statuses in (i) hub genes, (ii) gene pairs with small topological distances and (iii) gene pairs with high cumulative C-scores were explored. Finally, patients were stratified by tumor mutational burden high *versus* low to examine and compare RFS and OS differences using the ‘survival’ and ‘survminer’ packages in R (Version 3.6.2).

## Results

### TMB analysis

In the Vigil group, 33 patients had high TMB and 14 low TMB, with similar proportions in the placebo group (34 and 10, respectively). Within Vigil treated patients there was no impact of TMB on RFS (HR = 1.289 90% CI 0.639–2.602) or OS (HR = 1.266 90% CI 0.448–3.578). Comparison of high TMB effect involved in Vigil compared to placebo related to RFS (HR = 0.598 90% CI 0.354–1.011 *p* = 0.052) and OS (HR = 0.514 90% CI 0.252–1.051 *p* = 0.06) was not significant, but a trend to the benefit of Vigil was suggested. However, this could have been related to reduced placebo response in patients with high TMB. Low TMB did not impact RFS or OS in patients receiving Vigil compared to placebo (HR = 1.011 90% CI 0.405–2.524 and HR = 1.153 90% CI 0.244–5.461 respectively). High TMB also did not disrupt significance of *BRCA*wt correlation with RFS previously reported [17]. In *BRCA*wt TMB high patients median RFS was 13.7 months in Vigil treated patients compared to 8 months for placebo (HR = 0.427 90% CI 0.232–0.784). Similarly, overall survival in *BRCA*wt TMB high patients was not reached in Vigil patients compared to 41.4 months in placebo (HR = 0.416 90% CI 0.187–0.926; Table 1). RFS and OS could not be evaluated in the *BRCA*wt, low TMB group due to small sample size.

**Table 1:** TMB did not impact Vigil efficacy in BRCAwt patients.

	*BRCA*wt/High TMB	BRCAwt/Low TMB
	Vigil *n* = 33	Vigil *n* = 14
	Placebo *n* = 34	Placebo *n* = 10
OS	HR = 0.598 90% CI 0.354–1.011 *p* = 0.052	HR = 1.011 90% CI 0.405–2.524
RFS	HR = 0.514 90% CI 0.252–1.051 *p* = 0.06	HR = 1.153 90% CI 0.244–5.461

### Network construction and pathway enrichment analysis

**Fig. 1:**
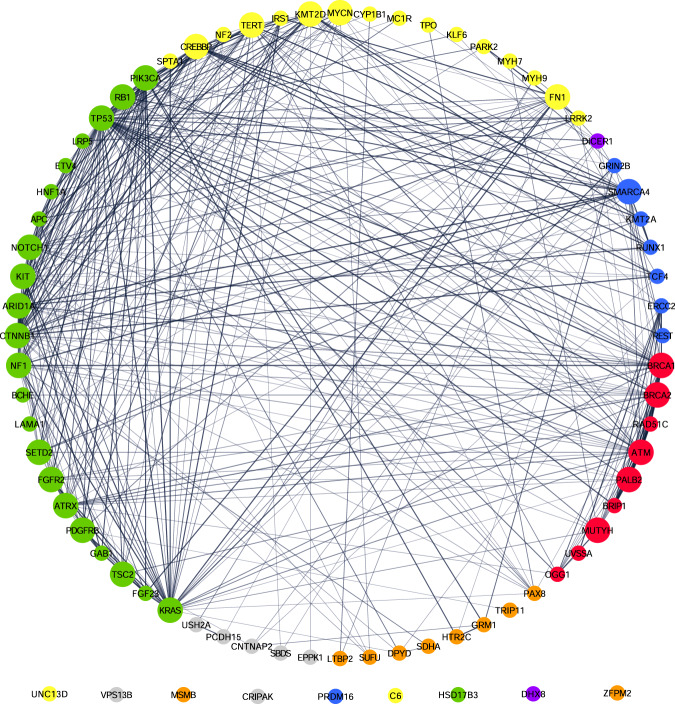
STRING Network produced in Cytoscape. Larger nodes indicate hub genes. A node colored red indicates a majority of pathways involved with DNA repair. Blue indicates chromosomal organization and transcription. Purple indicates regulation of translation and post-translational modification. Yellow indicates immunity. Green indicates other cancer genes. Gray genes are those which had no known pathways in WikiPathways orange genes do not fall into the other six categories.

To identify potential gene interactions that were associated with extended RFS in patients receiving Vigil, first a protein–protein interaction (PPI) network was constructed using 83 genes that were identified as having pathogenic mutations in the study population [42]. The 83 genes were loaded into STRING software; 77 of these genes were identified as having functional data in the database and were used to construct a network. The six genes that STRING did not recognize include AC092143.1, AL132855.1, MHRT, MYCN, NBR2 and ZFPM2-AS1. These were not included in the network or the degree chart. The STRING-constructed PPI network is displayed in Fig. 1. In the STRING network, an association or interaction may refer to direct (e.g., physical binding) or indirect interactions, such as shared participation in a common metabolic pathway [43]. Nodes in the network represent genes (*n* = 77) and edges (*n* = 371) represent biological interaction or association between any two of the identified pathogenic genes. Pathway analysis was conducted by inputting each gene in the STRING network into the WikiPathways Application in Cytoscape [42].

### Single gene analysis

#### Hub genes

**Fig. 2:**
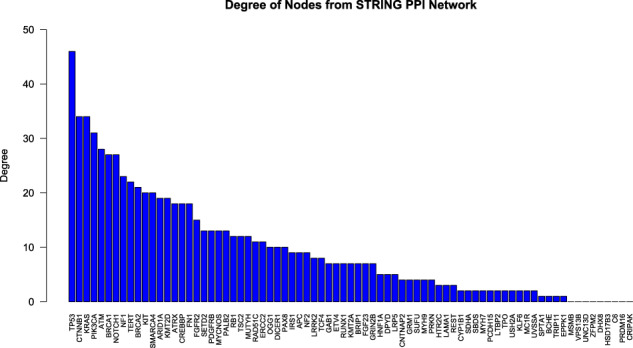
Degree of nodes from STRING PPI Network. Each gene in the STRING PPI Network is represented from largest to smallest degree below.

**Fig. 3:**
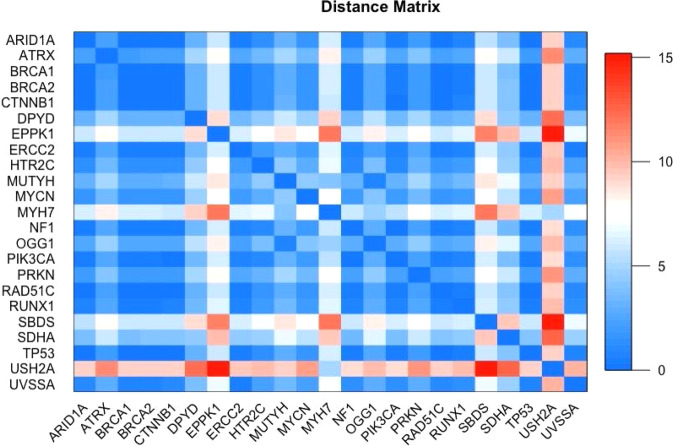
Distance matrix. Heat map key is located to the right of the matrix. Blue shading represents a high degree of interaction while red represents less interaction. The diagonal dark blue shade is a genes interaction with itself and is equal to 0.

A hub gene is defined to be a gene with a high degree (or number of connections) of associations to other genes in the network. Here, a hub gene is considered to be a gene with degree ≥12, which is the top quartile of genes based on the range of degree of genes in the network. Ten of the 23 genes in the distance matrix (Figure 3) were identified to have a degree ≥12, in the network defining them as hub genes: *TP53, CTNNB1, PIK3CA, BRCA1, NF1, BRCA2, ARID1A, ATRX, MYCNOS,* and *MUTYH. TP53* had the largest degree of all genes in the network as seen in Fig. 2.

For the single gene analysis, patients were grouped by their mutational status and the Kaplan–Meier and log-rank tests were performed to determine whether the mutational status at that loci was associated with RFS in Vigil treated patients relative to placebo. RFS from randomization was the primary endpoint of the VITAL trial and therefore it was used as the test variable (or categorical variable) for this analysis. *TP53*, *BRCA1*, and *BRCA2* were the only hub genes that reached our planned statistical cutoff of *p* ≤ 0.1 from randomization (Table 2). In patients with *TP53*-mutated tumors (*TP53*m; *n* = 65), median RFS was 18.69 months with Vigil (*n* = 33) and 8.35 months with placebo (*n* = 32) (one-sided *p* = 0.096, HR = 0.66). In the *BRCA1*wt population (*n* = 79), RFS was 12.75 months for Vigil (n = 42) and 8.38 months for placebo (*n* = 37) (one-sided *p* = 0.10, HR = 0.70). RFS for the *BRCA2*wt population (*n* = 82) was 11.47 months and 8.35 months for Vigil (*n* = 46) and placebo (*n* = 36), respectively (one-sided *p* = 0.05, HR = 0.64). Additional hub gene analysis is presented in Supplemental Table 1.

**Table 2:** RFS from randomization for *TP53m*, *BRCA1wt*, *and BRCA2wt*.

Gene	Vigil median RFS (months)	Placebo median RFS (months)	Difference (months)	*N*		*P*-value	HR
				Vigil	Placebo		
*TP53m*	18.69	8.35	10.35	33	32	0.096	0.66
*BRCA1*wt	12.75	8.38	4.37	42	37	0.10	0.70
*BRCA2*wt	11.47	8.35	3.12	46	36	0.05	0.64

### Gene pair analysis

#### Gene pairs with a small topological distance

The distance matrix only included 23 genes, representing a subset of the network that was complete (i.e., every pair of genes had an edge connecting them). Once the complete network was found, the distance matrix was constructed to visualize the topological distance of each corresponding gene pair.

The maximum and minimum distance in the matrix were between genes *USH2A* and *EPPK1*, with a distance of 15.19 and between genes *BRCA1* and *BRCA2* with a distance of 0.02, respectively. We further analyzed gene pairs with a small topological distance. Using a cutoff of 3.8 which was calculated by taking the range, 15.19 minus 0.02, and dividing by four, denoted the bottom quartile, we arrived at 139 gene pairs comprised of 23 genes (Fig. 3).

We found that *TP53* and *BRCA1* had a distance of 0.04 and *TP53* and *BRCA2* had a distance of 0.06. This indicated that there was strong evidence that *TP53* had a functional association with *BRCA1* and *BRCA2* and thus considered both *BRCA1* and *BRCA2* as a joint relationship designated as *BRCA* which is consistent with prior analysis by others [44]. Additional genes with small topological distance are presented in Supplemental Tables 2 and 3.

#### Gene pairs with high cumulative C-Scores

After computing the C-Score for all gene pairs in the Distance Matrix and ordering genes from highest to lowest cumulative C-Score (*cumC-score*), the genes with highest *cumC-score* in order were *BRCA1*, *BRCA2* and *TP53*. This indicated high connectivity within the network both from a co-mutation standpoint and a topological distance perspective. C-score significance analysis was performed, however due to the limitations of small sample size and small gene sets the results were not significant. Future analysis with larger sample size and gene panels is warranted.

**Fig. 4:**
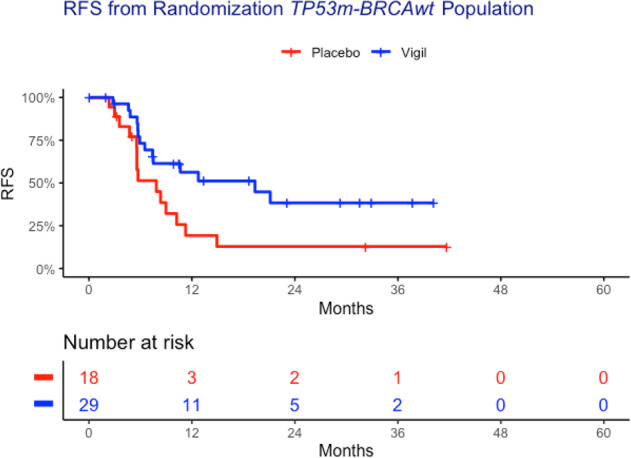
Kaplan–Meier curves of *TP53m-BRCAwt* population RFS from time of randomization. Vigil demonstrates RFS advantage (HR = 0.44, *p* = 0.01) in the *TP53m-BRCAwt* population.

The function or dysfunction of *BRCA1*, *BRCA2*, and *TP53* has broad-reaching consequences for the other proteins within the ovarian cancer cell, more so than other genomic variants. This warranted closer attention to the effects of wild type versus mutant expression. Given the proximity of *TP53* with *BRCA1* and *BRCA2* in the STRING network and their high cumulative C-scores, we performed survival analysis across the four mutation statuses (co-mutant, mutant-wild-type, wild-type mutant and co-wild type). The impact of these combinations on relapse-free survival in the Vigil treatment group compared to placebo is displayed in Table 3. The *TP53*m-*BRCA*wt group experienced a median RFS of 19.35 months, compared to 11.71 months in co-mutant and 10.48 months in co-wild type. When compared between treatment arms, the *TP53*m-*BRCA*wt group had a median RFS of 19.35 months in the Vigil arm compared to 7.85 months in placebo (*p* = 0.01, HR = 0.44; Fig. 4). Additional tests demonstrated statistical significance (*p* < 0.05) for the combination of *BRCA* and our identified hub genes, displayed in Table 4. Results regarding other gene pairs with small distances are presented in the Supplemental data.

**Table 3:** RFS from randomization for *TP53* and *BRCA*.

Gene 1	Gene 2	Vigil median RFS (months)	Placebo median RFS (months)	Difference (months)	*N*		*P*-value	HR
					Vigil	Placebo		
*BRCA*m	*TP53*m	11.71	14.75	−3.04	4	14	0.27	1.50
*BRCA*wt	*TP53*m	19.35	7.85	11.50	29	18	0.013	0.44
*BRCA*m	*TP53*wt	10.48	31.90	−21.42	3	3	0.39	1.40
*BRCA*wt	*TP53*wt	10.48	8.38	2.10	11	9	0.47	1.05

**Table 4:** RFS from randomization for *BRCAwt* and other hub genes of significance.

Gene 1	Gene 2	Vigil median RFS (months)	Placebo median RFS (months)	Difference (months)	*N*		*P*-value	HR
					Vigil	Placebo		
*BRCA*wt	*PIK3CA*wt	11.47	7.95	3.52	39	26	0.02	0.53
*BRCA*wt	*NF1*wt	12.75	8.35	4.40	40	26	0.04	0.59
*BRCA*wt	*ARID1A*wt	12.75	7.95	4.80	37	24	0.05	0.59
*BRCA*wt	*MYCNOS*wt	13.67	5.72	7.95	27	17	0.03	0.47
*BRCA*wt	*MUTYH*wt	12.75	7.95	4.80	38	27	0.03	0.56

#### Homologous recombination status

**Fig. 5:**
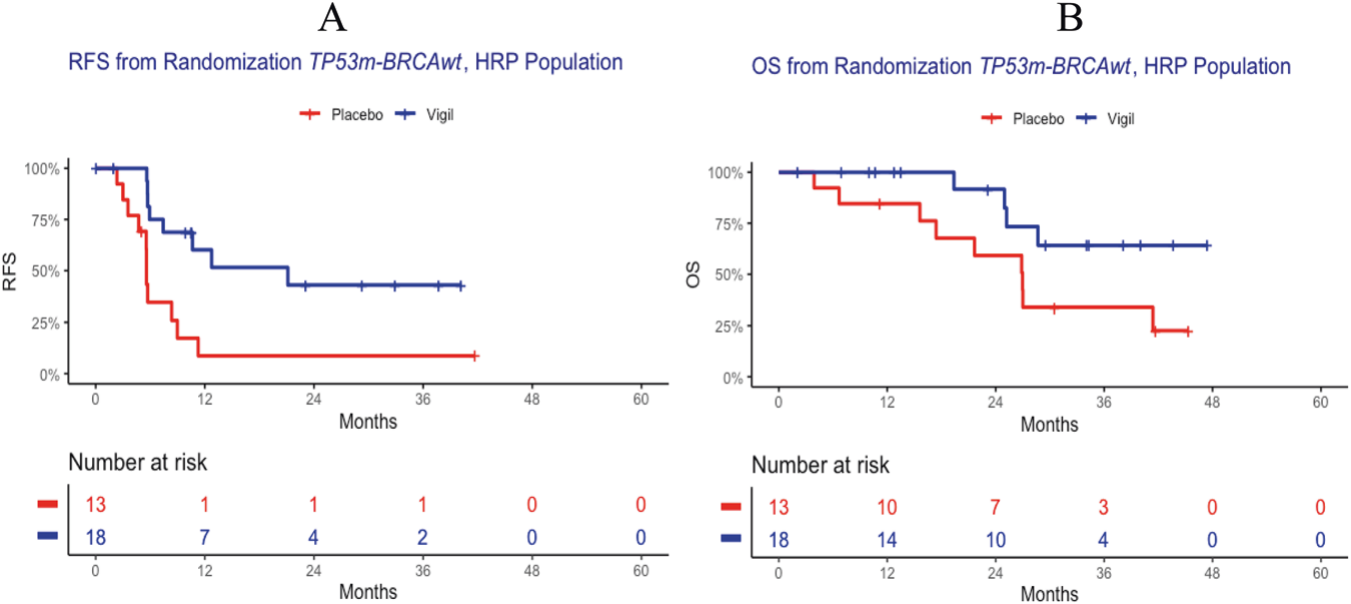
KM curves of *TP53m-BRCAwt*, HRP population from randomization. Vigil demonstrated RFS (HR = 0.26, *p* = 0.001) (**A**) and OS (HR = 0.33, *p* = 0.02) (**B**) from time of randomization in the *TP53m-BRCAwt*, HRP population.

KM analysis was conducted to determine the effect of homologous recombination status on the *TP53*m-*BRCA*wt population. A score of <42, as defined by Myriad Genetics was used to identify patients who were HRP and a score of ≥42 indicated patients were HRD. RFS in the *TP53*m-*BRCA*wt and HRP group was improved to 21.1 vs. 5.6 months (HR = 0.26, *p* = 0.001) in Vigil vs. placebo patients (Fig. 5A). OS was also improved in HRP and *TP53*m-*BRCA*wt patients from randomization. In the Vigil treated group, OS was not reached while placebo was 27.0 months (HR = 0.33, *p* = 0.02; Fig. 5B).

## Discussion

Our network-based analysis of pathogenic gene mutations points us towards a potential optimally responsive population to Vigil, specifically, patients with a HRP malignant cell profile including *BRCA*wt and *TP53* mutant gene signals. These results are only hypothesis generating, but suggest a novel methodological/computational approach to biomarker assessment and for optimizing a target population for Vigil therapy and possibly proof of principle for biomarker assessment of other target-based therapies. Further evaluation of other hub genes (*PIK3CA*wt, *NF1*wt, *ARID1*wt, *MYCNOS*wt, and *MUTYH*wt) in *BRCA1/2*wt, HRP cancer patients as potential biomarkers for Vigil treatment, and possibly indicators of novel added therapeutic management, may be fruitful. In our approach, the STRING database was utilized to construct an unbiased network to describe the functional similarity between genes, thereby providing mechanistic understanding of the potential effect of wild type or mutant variants. This approach circumvents a potential limitation of DNA variant data and may provide more effective target population identification, given our current limited understanding of comprehensive molecular signal expression pathways and relationship to clinical benefit impact. In this manner, one can describe the genes of high importance by computationally analyzing properties of the malignant network, such as the topological distance between genes, C-scores, and hub genes. Through these analyses in the HRP ovarian population treated with Vigil, three gene variants stood out across all analytic methods: *BRCA1*, *BRCA2*, and *TP53*. Similar methodology has been used by others involving various cancer types and have demonstrated molecular collaboration with clinical outcomes [40, 41, 43, 45].

The combination of STRING-generated topological distance and sample-derived probability of co-mutation is manifested as the C-score for two genes, and the *cum*C-score is the aggregate of one gene’s interaction with every other gene in the network. Gene pairs identified by C-scores often involve central cancer genes which correlate with increased tumorigenesis and sensitivity/resistance to anticancer therapeutics [41]. Due to limited size of our gene set and patient sample size, we chose to characterize individual genes by their *cum*C-score [43]. Here, we identified *TP53*, *BRCA1*, and *BRCA2* as genes with the highest *cumC*-scores of the genes present in patient samples. The high *cum*C-score suggests that particular variants of these genes correspond to drug response, as *cum*C-scores correlate with sensitivity and resistance [41]. Indeed, we found that *TP53*m and *BRCA*wt correlated with increased RFS benefit to Vigil, although further prospective analysis, now underway, will be required for verification. Additional topological features of the STRING network which may be of interest and worthy of further investigation include betweenness centrality (BC), eccentricity, closeness centrality, and eigenvector centrality. A node with a high BC may represent a bottleneck in the network [46]. This may also shed light on additional signal patterns and additional genes relevant to ovarian cancer and Vigil mechanism, which may be further validated through analysis of expression data in future study.

Analysis of hub genes similarly identified *BRCA* and *TP53* as central ovarian cancer genes. Previous work demonstrated that hub gene data provided clinical insight to differences in OS. In one previous study, 4 of 16 identified hub genes in the studied sample (*CCNB1*, *CENPF*, *KIF11*, and *ZWINT*) were associated with decreased OS of patients with ovarian cancer [47]. Authors of this study posit that mutations in these hub genes, which occupy the intersection of many cellular pathways, results in rippling dysregulation of numerous cellular functions. Thus, by altering a single hub gene, cellular homeostasis may be impacted on a larger scale. This disruption may be associated with tumor progression, immune inhibition, and any number of cancer hallmarks, which may explain the association of hub genes with a poor prognosis [48, 49]. The hub gene analysis presented in our paper identified *TP53*m, *BRCA1*wt, *BRCA2*wt as core hub genes with RFS advantage in Vigil treated patients, potentially indicating a broader genetic network for target population of Vigil. Moreover, this approach supports a strategic shift in targeted therapeutic development towards targeting related network genomic variants.

Our results also support that the pathways impacted by *BRCA* must be intact for Vigil to function optimally, while the pathways impacted by *TP53* may be dysregulated. Similarly, the integrity of the homologous repair pathway and its associated genes (HRP genotype) may also be important for optimal Vigil results. This suggests a cancer homeostasis formed by the combination of gene variants that creates an optimal environment for drug sensitivity or resistance. We hypothesize that the interaction of pathways generated by functional HR or *BRCA* proteins and disrupted *TP53* protein creates the ideal molecular setting for Vigil therapy responsiveness. Mutation in *TP53* is likely an early oncogenic event and likely results in clonal cell *TP53* neoantigen expression. Coupled with proficient homologous recombination of *BRCA1/2* wild-type gene patients may achieve low TMB, but also low intratumor heterogeneity (ITH). While low TMB potentially results in decreased CD4+/CD8+ T cells infiltration, this signaling pattern and its associated low ITH may provide for more effective and consolidated T cell response towards clonal neoantigens. Furthermore, treatment with Vigil may bypass the effect of low TMB by increasing circulating CD4+/CD8+ T cells and providing T cell neoantigen education, which would likely be particularly effective in this population [20]. Conversely, ITH associated with high TMB provides an increase in variation of neoepitopes between cells within a tumor and can dilute a consolidated immune response against lower frequency clonal neoantigens [17, 28]. As related to Vigil mechanism of action a more robust immune attack is mounted against clonal neoepitopes displayed by the majority of cancer cells in a low ITH environment. *TP53*m is associated with increased TMB and tumor aneuploidy level (TAL), which have conflicting impacts on immune responsivity. TMB tends to correlate with sensitivity to certain immunotherapies particularly checkpoint inhibitors [50, 51]. TAL is the degree of chromosomal mis-segregation, and is associated with poor response to immunotherapy [52, 53]. *TP53*’s impact on immunogenicity is also tissue dependent, which may be determined by differential gene expression within tissue types [53–55]. This phenomenon may further explain mutant *TP53* genotype’s association with improved response to Vigil immunotherapy in the ovarian cancer *BRCA*wt population. We did not observe an independent beneficial effect of high TMB with Vigil although evidence of detrimental effect of high TMB was suggested in placebo patients, and reported by others [56]. Although previously reported benefit in RFS and OS in *BRCA*wt patients was not adversely effected by TMB [17].

In conclusion, despite sample size limitation, we demonstrate proof of support for use of DNA analytical methods to separate resistant and sensitive populations to Vigil. These techniques create a robust approach to analyze how the nodal network relationship between genes affects clinical response to Vigil when used as maintenance therapy in advanced stage III/IV resectable disease patients. These results are hypothesis generating and warrant further investigation. Moreover, these results further support novel use of network-based analysis to identify other more sensitive gene targets and potentially additional novel targeted therapeutic combinations with Vigil and possibly other immunotherapeutics.

## Supplementary information

Supplemental Material

## Data Availability

Data can be shared following an approved request for a specific research question. Requests may be declined by Gradalis, Inc if deemed to pose a conflict of interest or competitive risk.

## References

[1] Bray F, Ferlay J, Soerjomataram I, Siegel RL, Torre LA, Jemal A (2018). Global cancer statistics 2018: GLOBOCAN estimates of incidence and mortality worldwide for 36 cancers in 185 countries. CA Cancer J Clin.

[2] Coburn SB, Bray F, Sherman ME, Trabert B (2017). International patterns and trends in ovarian cancer incidence, overall and by histologic subtype. Int J Cancer.

[3] Momenimovahed Z, Tiznobaik A, Taheri S, Salehiniya H (2019). Ovarian cancer in the world: epidemiology and risk factors. Int J Women’s Health.

[4] Yoneda A, Lendorf ME, Couchman JR, Multhaupt HA (2012). Breast and ovarian cancers: a survey and possible roles for the cell surface heparan sulfate proteoglycans. J Histochem Cytochem.

[5] Walsh T, Casadei S, Lee MK, Pennil CC, Nord AS, Thornton AM (2011). Mutations in 12 genes for inherited ovarian, fallopian tube, and peritoneal carcinoma identified by massively parallel sequencing. Proc Natl Acad Sci USA.

[6] Toss A, Tomasello C, Razzaboni E, Contu G, Grandi G, Cagnacci A (2015). Hereditary ovarian cancer: not only BRCA 1 and 2 genes. Biomed Res Int.

[7] Cortez AJ, Tudrej P, Kujawa KA, Lisowska KM (2018). Advances in ovarian cancer therapy. Cancer Chemother Pharmacol.

[8] Jelovac D, Armstrong DK (2011). Recent progress in the diagnosis and treatment of ovarian cancer. CA Cancer J Clin.

[9] Foley OW, Rauh-Hain JA, del Carmen MG (2013). Recurrent epithelial ovarian cancer: an update on treatment. Oncology.

[10] Ledermann J, Harter P, Gourley C, Friedlander M, Vergote I, Rustin G (2012). Olaparib maintenance therapy in platinum-sensitive relapsed ovarian cancer. New Engl J Med.

[11] Gibson BA, Kraus WL (2012). New insights into the molecular and cellular functions of poly(ADP-ribose) and PARPs. Nat Rev Mol Cell Biol.

[12] Faraoni I, Graziani G (2018). Role of BRCA mutations in cancer treatment with poly(ADP-ribose) polymerase (PARP) inhibitors. Cancers.

[13] Kaelin WG (2005). The concept of synthetic lethality in the context of anticancer therapy. Nat Rev Cancer.

[14] Chan N, Pires IM, Bencokova Z, Coackley C, Luoto KR, Bhogal N (2010). Contextual synthetic lethality of cancer cell kill based on the tumor microenvironment. Cancer Res.

[15] Gonzalez-Martin A, Pothuri B, Vergote I, DePont Christensen R, Graybill W, Mirza MR (2019). Niraparib in patients with newly diagnosed advanced ovarian cancer. New Engl J Med.

[16] Ray-Coquard I, Pautier P, Pignata S, Perol D, Gonzalez-Martin A, Berger R (2019). Olaparib plus bevacizumab as first-line maintenance in ovarian cancer. New Engl J Med.

[17] Rocconi RP, Grosen E, Ghamande SA, Chan JK, Barve M, Oh J (2020). Gemogenovatucel-T (Vigil) immunotherapy as maintenance in frontline stage III/IV ovarian cancer (VITAL): a randomised, double-blind, placebo-controlled, phase 2b trial. Lancet Oncol.

[18] Senzer N, Barve M, Nemunaitis J, Kuhn J, Melnyk A, Beitsch P (2013). Long term follow up: phase I trial of “bi-shRNA furin/GMCSF DNA/autologous tumor cell” immunotherapy (FANG™) in advanced cancer. J Vaccines Vaccin.

[19] Oh J, Barve M, Matthews CM, Koon EC, Heffernan TP, Fine B (2016). Phase II study of Vigil(R) DNA engineered immunotherapy as maintenance in advanced stage ovarian cancer. Gynecol Oncol.

[20] Herron J, Smith N, Stanbery L, Aaron P, Manning L, Bognar E (2020). Vigil: personalized immunotherapy generating systemic cytotoxic T cell response. Cancer Sci Res.

[21] Rocconi RP, Stanbery L, Madeira da Silva L (2021). Long-Term Follow-Up of Gemogenovatucel-T (Vigil) Survival and Molecular Signals of Immune Response in Recurrent Ovarian Cancer. Vaccines (Basel).

[22] Barve M, Kuhn J, Lamont J, Beitsch P, Manning L, O Pappen B (2016). Follow-up of bi-shRNA furin/GM-CSF Engineered Autologous Tumor Cell (EATC) Immunotherapy Vigilr® in patients with advanced melanoma. Biomed. Genet. Genom.

[23] Ghisoli M, Barve M, Mennel R, Lenarsky C, Horvath S, Wallraven G (2016). Three-year Follow up of GMCSF/bi-shRNA(furin) DNA-transfected Autologous Tumor Immunotherapy (Vigil) in Metastatic Advanced Ewing’s Sarcoma. Mol Ther.

[24] Ghisoli M, Barve M, Schneider R, Mennel R, Lenarsky C, Wallraven G (2015). Pilot trial of FANG immunotherapy in Ewing’s sarcoma. Mol Ther.

[25] Oh J, Barve M, Senzer N, Aaron P, Manning L, Wallraven G (2020). Long-term follow-up of Phase 2A trial results involving advanced ovarian cancer patients treated with Vigil(R) in frontline maintenance. Gynecol Oncol Rep.

[26] Senzer N, Barve M, Kuhn J, Melnyk A, Beitsch P, Lazar M (2012). Phase I trial of “bi-shRNAi(furin)/GMCSF DNA/autologous tumor cell” vaccine (FANG) in advanced cancer. Mol Ther.

[27] Rocconi RP, Monk BJ, Walter A, Herzog TJ, Galanis E, Manning L (2021). Gemogenovatucel-T (Vigil) immunotherapy demonstrates clinical benefit in homologous recombination proficient (HRP) ovarian cancer. Gynecol Oncol.

[28] McGranahan N, Furness AJ, Rosenthal R, Ramskov S, Lyngaa R, Saini SK (2016). Clonal neoantigens elicit T cell immunoreactivity and sensitivity to immune checkpoint blockade. Science.

[29] Landrum MJ, Lee JM, Benson M, Brown GR, Chao C, Chitipiralla S (2018). ClinVar: improving access to variant interpretations and supporting evidence. Nucleic Acids Res.

[30] Vanderwalde A, Spetzler D, Xiao N, Gatalica Z, Marshall J (2018). Microsatellite instability status determined by next-generation sequencing and compared with PD-L1 and tumor mutational burden in 11,348 patients. Cancer Med.

[31] Szklarczyk D, Gable AL, Lyon D, Junge A, Wyder S, Huerta-Cepas J (2019). STRING v11: protein-protein association networks with increased coverage, supporting functional discovery in genome-wide experimental datasets. Nucleic Acids Res.

[32] Szklarczyk D, Morris JH, Cook H, Kuhn M, Wyder S, Simonovic M (2017). The STRING database in 2017: quality-controlled protein-protein association networks, made broadly accessible. Nucleic Acids Res.

[33] Szklarczyk D, Franceschini A, Wyder S, Forslund K, Heller D, Huerta-Cepas J (2015). STRING v10: protein-protein interaction networks, integrated over the tree of life. Nucleic Acids Res.

[34] Franceschini A, Szklarczyk D, Frankild S, Kuhn M, Simonovic M, Roth A (2013). STRING v9.1: protein-protein interaction networks, with increased coverage and integration. Nucleic Acids Res.

[35] Szklarczyk D, Franceschini A, Kuhn M, Simonovic M, Roth A, Minguez P (2011). The STRING database in 2011: functional interaction networks of proteins, globally integrated and scored. Nucleic Acids Res.

[36] Jensen LJ, Kuhn M, Stark M, Chaffron S, Creevey C, Muller J (2009). STRING 8-a global view on proteins and their functional interactions in 630 organisms. Nucleic Acids Res.

[37] von Mering C, Jensen LJ, Kuhn M, Chaffron S, Doerks T, Krüger B (2007). STRING 7-recent developments in the integration and prediction of protein interactions. Nucleic Acids Res.

[38] von Mering C, Huynen M, Jaeggi D, Schmidt S, Bork P, Snel B (2003). STRING: a database of predicted functional associations between proteins. Nucleic Acids Res.

[39] Snel B, Lehmann G, Bork P, Huynen MA (2000). STRING: a web-server to retrieve and display the repeatedly occurring neighbourhood of a gene. Nucleic Acids Res.

[40] Li BQ, Huang T, Liu L, Cai YD, Chou KC (2012). Identification of colorectal cancer related genes with mRMR and shortest path in protein-protein interaction network. PLoS ONE.

[41] Liu C, Zhao J, Lu W, Dai Y, Hockings J, Zhou Y (2020). Individualized genetic network analysis reveals new therapeutic vulnerabilities in 6,700 cancer genomes. PLoS Comput Biol.

[42] Shannon P, Markiel A, Ozier O, Baliga NS, Wang JT, Ramage D (2003). Cytoscape: a software environment for integrated models of biomolecular interaction networks. Genome Res.

[43] von Mering C, Jensen LJ, Snel B, Hooper SD, Krupp M, Foglierini M (2005). STRING: known and predicted protein-protein associations, integrated and transferred across organisms. Nucleic Acids Res.

[44] Li Y, Zhou X, Liu J, Yin Y, Yuan X, Yang R (2020). Differentially expressed genes and key molecules of BRCA1/2-mutant breast cancer: evidence from bioinformatics analyses. PeerJ.

[45] Franceschini A, Lin J, von Mering C, Jensen LJ (2016). SVD-phy: improved prediction of protein functional associations through singular value decomposition of phylogenetic profiles. Bioinformatics.

[46] Chen SJ, Liao DL, Chen CH, Wang TY, Chen KC (2019). Construction and analysis of protein-protein interaction network of heroin use disorder. Sci Rep.

[47] Xu Z, Zhou Y, Cao Y, Dinh TL, Wan J, Zhao M (2016). Identification of candidate biomarkers and analysis of prognostic values in ovarian cancer by integrated bioinformatics analysis. Med Oncol.

[48] Hanahan D, Weinberg RA (2011). Hallmarks of cancer: the next generation. Cell.

[49] Hanahan D, Weinberg RA (2000). The hallmarks of cancer. Cell.

[50] Yarchoan M, Albacker LA, Hopkins AC, Montesion M, Murugesan K, Vithayathil TT (2019). PD-L1 expression and tumor mutational burden are independent biomarkers in most cancers. JCI Insight.

[51] Choucair K, Morand S, Stanbery L, Edelman G, Dworkin L, Nemunaitis J (2020). TMB: a promising immune-response biomarker, and potential spearhead in advancing targeted therapy trials. Cancer Gene Ther.

[52] Davoli T, Uno H, Wooten EC, Elledge SJ (2017). Tumor aneuploidy correlates with markers of immune evasion and with reduced response to immunotherapy. Science.

[53] Li L, Li M, Wang X (2020). Cancer type-dependent correlations between TP53 mutations and antitumor immunity. DNA Repair.

[54] Andrysik Z, Galbraith MD, Guarnieri AL, Zaccara S, Sullivan KD, Pandey A (2017). Identification of a core TP53 transcriptional program with highly distributed tumor suppressive activity. Genome Res.

[55] Kastenhuber ER, Lowe SW (2017). Putting p53 in context. Cell.

[56] Shao C, Li G, Huang L, Pruitt S, Castellanos E, Frampton G (2020). Prevalence of high tumor mutational burden and association with survival in patients with less common solid tumors. JAMA Netw Open.

