# Supplementary material for: Network based analysis identifies TP53m-BRCA1/2wt-homologous recombination proficient (HRP) population with enhanced susceptibility to Vigil immunotherapy
Source: Cancer Gene Ther. 2021 Nov 16;29(7):993–1000. doi: 10.1038/s41417-021-00400-x (PMC9293751; doi:10.1038/s41417-021-00400-x)
Supplement: Supplementary file 1 — Supplemental Material [file 41417_2021_400_MOESM1_ESM.docx]

**Supplemental Materials**

**Figure 1 Mutation Matrix**

**Table 1. RFS From Procurement – Hub Genes**

| **Gene** | **VigilMedian** | **PlaceboMedian** | **Difference** | **nVigil** | **nPlacebo** | **PVAL** |
| --- | --- | --- | --- | --- | --- | --- |
| PIK3CAwt | 18.2998 | 15.8686 | 2.4312 | 46 | 43 | 0.0799 |
| * PIK3CAm was unable to be performed due to small sample size | | | | | | |

| **Gene** | **VigilMedian** | **PlaceboMedian** | **Difference** | **nVigil** | **nPlacebo** | **PVAL** |
| --- | --- | --- | --- | --- | --- | --- |
| NF1wt | 18.3326 | 16 | 2.3326 | 47 | 42 | 0.0742 |
| * NF1m was unable to be performed due to small sample size | | | | | | |

| **Gene** | **VigilMedian** | **PlaceboMedian** | **Difference** | **nVigil** | **nPlacebo** | **PVAL** |
| --- | --- | --- | --- | --- | --- | --- |
| BRCA2wt | 18.2998 | 14.9158 | 3.384 | 46 | 36 | 0.0304 |
| * BRCA2m was unable to be performed due to small sample size | | | | | | |

| **Gene** | **VigilMedian** | **PlaceboMedian** | **Difference** | **nVigil** | **nPlacebo** | **PVAL** |
| --- | --- | --- | --- | --- | --- | --- |
| ARID1Am | 17.9713 | 14.3573 | 3.614 | 3 | 4 | 0.0365 |
| ARID1Awt | 18.3326 | 16 | 2.3326 | 44 | 40 | 0.1142 |

| **Gene** | **VigilMedian** | **PlaceboMedian** | **Difference** | **nVigil** | **nPlacebo** | **PVAL** |
| --- | --- | --- | --- | --- | --- | --- |
| MUTYHwt | 18.3326 | 16 | 2.3326 | 45 | 44 | 0.0879 |
| * MUTYHm was unable to be performed due to small sample size | | | | | | |

**Table 2. RFS From Randomization – Small Distance Gene Pairs**

| **Gene 1** | **Gene 2** | **VigilMedian** | **PlaceboMedian** | **Difference** | **nVigil** | **nPlacebo** | **PVAL** |
| --- | --- | --- | --- | --- | --- | --- | --- |
| UVSSAm | ATRXm | NR | 11.7947 | NR | 9 | 6 | 0.2556 |
| UVSSAwt | ATRXm | 11.4661 | 8.3778 | 3.0883 | 32 | 28 | 0.359 |
| UVSSAwt | ATRXwt | NR | 8.4928 | NR | 4 | 4 | 0.0386 |
| *UVSSAm – ATRXwt was unable to be performed due to small sample size  * NR indicates not reached | | | | | | | |

| **Gene 1** | **Gene 2** | **VigilMedian** | **PlaceboMedian** | **Difference** | **nVigil** | **nPlacebo** | **PVAL** |
| --- | --- | --- | --- | --- | --- | --- | --- |
| BRCA2wt | BRCA1m | 4.731 | 11.4004 | -6.6694 | 5 | 6 | 0.4507 |
| BRCA2wt | BRCA1wt | 11.4661 | 8.345 | 3.1211 | 41 | 30 | 0.0354 |
| *BRCA2m-BRCA1m and BRCA2m-BRCA1wt were unable to be performed due to small sample size | | | | | | | |

| **Gene 1** | **Gene 2** | **VigilMedian** | **PlaceboMedian** | **Difference** | **nVigil** | **nPlacebo** | **PVAL** |
| --- | --- | --- | --- | --- | --- | --- | --- |
| ERCC2wt | BRCA2wt | 11.4661 | 8.345 | 3.1211 | 46 | 34 | 0.0566 |
| *ERCC2m – BRCA2m, ERCC2m – BRCA2wt, ERCC2wt – BRCA2m were unable to be performed due to small sample size | | | | | | | |

| **Gene 1** | **Gene 2** | **VigilMedian** | **PlaceboMedian** | **Difference** | **nVigil** | **nPlacebo** | **PVAL** |
| --- | --- | --- | --- | --- | --- | --- | --- |
| MUTYHwt | BRCA2wt | 11.4661 | 8.345 | 3.1211 | 44 | 36 | 0.0458 |
| *MUTYHm – BRCA2m, MUTYHm – BRCA2wt, MUTYHwt – BRCA2m were unable to be performed due to small sample size | | | | | | | |

| **Gene 1** | **Gene 2** | **VigilMedian** | **PlaceboMedian** | **Difference** | **nVigil** | **nPlacebo** | **PVAL** |
| --- | --- | --- | --- | --- | --- | --- | --- |
| OGG1wt | BRCA2wt | 11.4661 | 8.345 | 3.1211 | 45 | 35 | 0.0552 |
| * OGG1m – BRCA2m, OGG1m – BRCA2wt, OGG1wt – BRCA2m were unable to be performed due to small sample size | | | | | | | |

| **Gene 1** | **Gene 2** | **VigilMedian** | **PlaceboMedian** | **Difference** | **nVigil** | **nPlacebo** | **PVAL** |
| --- | --- | --- | --- | --- | --- | --- | --- |
| PIK3CAwt | BRCA2wt | 10.6448 | 8.0493 | 2.5955 | 45 | 35 | 0.0351 |
| * PIK3CAm – BRCA2m, PIK3CAm – BRCA2wt, PIK3CAwt – BRCA2m were unable to be performed due to small sample size | | | | | | | |

| **Gene 1** | **Gene 2** | **VigilMedian** | **PlaceboMedian** | **Difference** | **nVigil** | **nPlacebo** | **PVAL** |
| --- | --- | --- | --- | --- | --- | --- | --- |
| PRKNwt | BRCA2wt | 11.4661 | 8.0493 | 3.4168 | 44 | 35 | 0.0357 |
| * PRKNm – BRCA2m, PRKNm – BRCA2wt, PRKNwt – BRCA2m were unable to be performed due to small sample size | | | | | | | |

| **Gene 1** | **Gene 2** | **VigilMedian** | **PlaceboMedian** | **Difference** | **nVigil** | **nPlacebo** | **PVAL** |
| --- | --- | --- | --- | --- | --- | --- | --- |
| RAD51Cwt | BRCA2wt | 11.4661 | 8.345 | 3.1211 | 45 | 35 | 0.0464 |
| * RAD51m – BRCA2m, RAD51m – BRCA2wt, RAD51wt – BRCA2m were unable to be performed due to small sample size | | | | | | | |

| **Gene 1** | **Gene 2** | **VigilMedian** | **PlaceboMedian** | **Difference** | **nVigil** | **nPlacebo** | **PVAL** |
| --- | --- | --- | --- | --- | --- | --- | --- |
| RUNX1wt | BRCA2wt | 11.4661 | 8.0493 | 3.4168 | 45 | 35 | 0.0379 |
| * RUNX1m – BRCA2m, RUNX1m – BRCA2wt, RUNX1wt – BRCA2m were unable to be performed due to small sample size | | | | | | | |

| **Gene 1** | **Gene 2** | **VigilMedian** | **PlaceboMedian** | **Difference** | **nVigil** | **nPlacebo** | **PVAL** |
| --- | --- | --- | --- | --- | --- | --- | --- |
| TP53m | ERCC2wt | 18.694 | 8.345 | 10.3491 | 33 | 30 | 0.0577 |
| TP53wt | ERCC2wt | 10.4805 | 19.614 | -9.1335 | 14 | 11 | 0.432 |
| * TP53m – ERCC2m and TP53wt – ERCC2m were unable to be performed due to small sample size | | | | | | | |

| **Gene 1** | **Gene 2** | **VigilMedian** | **PlaceboMedian** | **Difference** | **nVigil** | **nPlacebo** | **PVAL** |
| --- | --- | --- | --- | --- | --- | --- | --- |
| TP53m | HTR2Cm | 12.7474 | 5.7823 | 6.9651 | 10 | 3 | 0.0395 |
| TP53m | HTR2Cwt | 18.694 | 8.9692 | 9.7248 | 23 | 29 | 0.213 |
| TP53wt | HTR2Cwt | 13.6674 | 8.1643 | 5.5031 | 7 | 10 | 0.3037 |
| *TP53wt – HTR2Cm was unable to be performed due to small sample size | | | | | | | |

| **Gene 1** | **Gene 2** | **VigilMedian** | **PlaceboMedian** | **Difference** | **nVigil** | **nPlacebo** | **PVAL** |
| --- | --- | --- | --- | --- | --- | --- | --- |
| TP53m | MYCNm | NR | 8.5092 | NR | 7 | 8 | 0.0592 |
| TP53m | MYCNwt | 18.694 | 8.345 | 10.3491 | 26 | 24 | 0.3284 |
| TP53wt | MYCNm | 5.7823 | 13.9959 | -8.2136 | 6 | 4 | 0.1119 |
| TP53wt | MYCNwt | 13.6674 | 13.8973 | -0.23 | 8 | 8 | 0.2458 |
| * NR indicates not reached | | | | | | | |

**Table 3. RFS From Procurement – Small Distance Gene Pairs**

| **Gene 1** | **Gene 2** | **VigilMedian** | **PlaceboMedian** | **Difference** | **nVigil** | **nPlacebo** | **PVAL** |
| --- | --- | --- | --- | --- | --- | --- | --- |
| BRCA1m | ARID1Awt | 17.577 | 16 | 1.577 | 5 | 6 | 0.4216 |
| BRCA1wt | ARID1Am | 17.9713 | 14.3573 | 3.614 | 3 | 3 | 0.0294 |
| BRCA1wt | ARID1Awt | 19.3511 | 15.8686 | 3.4825 | 39 | 34 | 0.1117 |
| *BRCA1m – ARID1Am was unable to be performed due to small sample size | | | | | | | |

| **Gene 1** | **Gene 2** | **VigilMedian** | **PlaceboMedian** | **Difference** | **nVigil** | **nPlacebo** | **PVAL** |
| --- | --- | --- | --- | --- | --- | --- | --- |
| BRCA2wt | ARID1Am | 17.9713 | 14.3573 | 3.614 | 3 | 3 | 0.0294 |
| BRCA2wt | ARID1Awt | 18.3326 | 15.8686 | 2.4641 | 43 | 33 | 0.0476 |
| *BRCA2m-BARID1Am and BRCA2m-ARID1Awt were unable to be performed due to small sample size | | | | | | | |

| **Gene 1** | **Gene 2** | **VigilMedian** | **PlaceboMedian** | **Difference** | **nVigil** | **nPlacebo** | **PVAL** |
| --- | --- | --- | --- | --- | --- | --- | --- |
| CTNNB1wt | ARID1Am | 17.9713 | 14.3573 | 3.614 | 3 | 4 | 0.0365 |
| CTNNB1wt | ARID1Awt | 18.2998 | 16 | 2.2998 | 42 | 40 | 0.2056 |
| * CTNNB1m-ARID1Am and CTNNB1m-ARID1Awt were unable to be performed due to small sample size | | | | | | | |

| **Gene 1** | **Gene 2** | **VigilMedian** | **PlaceboMedian** | **Difference** | **nVigil** | **nPlacebo** | **PVAL** |
| --- | --- | --- | --- | --- | --- | --- | --- |
| ERCC2wt | ARID1Am | 17.9713 | 14.3573 | 3.614 | 3 | 4 | 0.0365 |
| ERCC2wt | ARID1Awt | 18.3326 | 16 | 2.3326 | 44 | 37 | 0.0853 |
| * ERCC2m-ARID1Am and ERCC2m-ARID1Awt were unable to be performed due to small sample size | | | | | | | |

| **Gene 1** | **Gene 2** | **VigilMedian** | **PlaceboMedian** | **Difference** | **nVigil** | **nPlacebo** | **PVAL** |
| --- | --- | --- | --- | --- | --- | --- | --- |
| HTR2Cm | ARID1Awt | 18.2998 | 17.7741 | 0.5257 | 17 | 5 | 0.2549 |
| HTR2Cwt | ARID1Am | 17.9713 | 14.3573 | 3.614 | 3 | 4 | 0.0365 |
| HTR2Cwt | ARID1Awt | 25.4949 | 16 | 9.4949 | 27 | 35 | 0.1505 |
| *HTR2Cm – ARID1Am was unable to be performed due to small sample size | | | | | | | |

| **Gene 1** | **Gene 2** | **VigilMedian** | **PlaceboMedian** | **Difference** | **nVigil** | **nPlacebo** | **PVAL** |
| --- | --- | --- | --- | --- | --- | --- | --- |
| MUTYHwt | ARID1Am | 17.9713 | 14.3573 | 3.614 | 3 | 4 | 0.0365 |
| MUTYHwt | ARID1Awt | 19.3511 | 16 | 3.3511 | 42 | 40 | 0.1117 |
| * MUTYHm-ARID1Am and MUTYHm-ARID1Awt were unable to be performed due to small sample size | | | | | | | |

| **Gene 1** | **Gene 2** | **VigilMedian** | **PlaceboMedian** | **Difference** | **nVigil** | **nPlacebo** | **PVAL** |
| --- | --- | --- | --- | --- | --- | --- | --- |
| NF1wt | ARID1Am | 17.9713 | 14.3573 | 3.614 | 3 | 4 | 0.0365 |
| NF1wt | ARID1Awt | 18.3326 | 16 | 2.3326 | 44 | 38 | 0.0971 |
| * NF1m-ARID1Am and NF1m-ARID1Awt were unable to be performed due to small sample size | | | | | | | |

| **Gene 1** | **Gene 2** | **VigilMedian** | **PlaceboMedian** | **Difference** | **nVigil** | **nPlacebo** | **PVAL** |
| --- | --- | --- | --- | --- | --- | --- | --- |
| PIK3CAwt | ARID1Am | 17.9713 | 14.3573 | 3.614 | 3 | 4 | 0.0365 |
| PIK3CAwt | ARID1Awt | 18.3326 | 16 | 2.3326 | 43 | 39 | 0.1043 |
| * PIK3CAm-ARID1Am and PIK3CAm-ARID1Awt were unable to be performed due to small sample size | | | | | | | |

| **Gene 1** | **Gene 2** | **VigilMedian** | **PlaceboMedian** | **Difference** | **nVigil** | **nPlacebo** | **PVAL** |
| --- | --- | --- | --- | --- | --- | --- | --- |
| PRKNwt | ARID1Am | 17.9713 | 14.3573 | 3.614 | 3 | 4 | 0.0365 |
| PRKNwt | ARID1Awt | 18.3326 | 16 | 2.3326 | 42 | 39 | 0.1065 |
| * PRKNm-ARID1Am and PRKNm-ARID1Awt were unable to be performed due to small sample size | | | | | | | |

| **Gene 1** | **Gene 2** | **VigilMedian** | **PlaceboMedian** | **Difference** | **nVigil** | **nPlacebo** | **PVAL** |
| --- | --- | --- | --- | --- | --- | --- | --- |
| RAD51Cwt | ARID1Am | 17.9713 | 14.3573 | 3.614 | 3 | 4 | 0.0365 |
| RAD51Cwt | ARID1Awt | 19.3511 | 17.7741 | 1.577 | 43 | 39 | 0.1094 |
| * RAD51Cm-RARID1Am and RAD51Cm-ARID1Awt were unable to be performed due to small sample size | | | | | | | |

| **Gene 1** | **Gene 2** | **VigilMedian** | **PlaceboMedian** | **Difference** | **nVigil** | **nPlacebo** | **PVAL** |
| --- | --- | --- | --- | --- | --- | --- | --- |
| RUNX1wt | ARID1Am | 17.9713 | 14.3573 | 3.614 | 3 | 4 | 0.0365 |
| RUNX1wt | ARID1Awt | 19.3511 | 16 | 3.3511 | 43 | 39 | 0.0942 |
| * RUNX1m-ARID1Am and RUNX1m-ARID1Awt were unable to be performed due to small sample size | | | | | | | |

| **Gene 1** | **Gene 2** | **VigilMedian** | **PlaceboMedian** | **Difference** | **nVigil** | **nPlacebo** | **PVAL** |
| --- | --- | --- | --- | --- | --- | --- | --- |
| UVSSAm | ARID1Awt | 17.577 | 17.7741 | -0.1971 | 11 | 11 | 0.1999 |
| UVSSAwt | ARID1Am | 17.9713 | 14.3573 | 3.614 | 3 | 3 | 0.0294 |
| UVSSAwt | ARID1Awt | 19.3511 | 15.8686 | 3.4825 | 33 | 29 | 0.1786 |
| * UVSSAm-ARID1Am was unable to be performed due to small sample size | | | | | | | |

| **Gene 1** | **Gene 2** | **VigilMedian** | **PlaceboMedian** | **Difference** | **nVigil** | **nPlacebo** | **PVAL** |
| --- | --- | --- | --- | --- | --- | --- | --- |
| BRCA2wt | ATRXm | 18.3326 | 15.8686 | 2.4641 | 40 | 28 | 0.1386 |
| BRCA2wt | ATRXwt | 17.9713 | 12.3532 | 5.6181 | 6 | 8 | 0.0373 |
| * BRCA2m-ATRXm and BRCA2m-ATRXwt were unable to be performed due to small sample size | | | | | | | |

| **Gene 1** | **Gene 2** | **VigilMedian** | **PlaceboMedian** | **Difference** | **nVigil** | **nPlacebo** | **PVAL** |
| --- | --- | --- | --- | --- | --- | --- | --- |
| NF1wt | ATRXm | 18.3326 | 16 | 2.3326 | 41 | 33 | 0.2374 |
| NF1wt | ATRXwt | 17.9713 | 12.4846 | 5.4867 | 6 | 9 | 0.0505 |
| * NF1m-ATRXm and NF1m-ATRXwt were unable to be performed due to small sample size | | | | | | | |

| **Gene 1** | **Gene 2** | **VigilMedian** | **PlaceboMedian** | **Difference** | **nVigil** | **nPlacebo** | **PVAL** |
| --- | --- | --- | --- | --- | --- | --- | --- |
| UVSSAm | ATRXm | NR | 18.7269 | NR | 9 | 6 | 0.1777 |
| UVSSAwt | ATRXm | 18.3326 | 14.9158 | 3.4168 | 32 | 28 | 0.3346 |
| UVSSAwt | ATRXwt | NR | 15.1786 | NR | 4 | 4 | 0.0386 |
| * UVSSAm-ATRXwt was unable to be performed due to small sample size | | | | | | | |

| **Gene 1** | **Gene 2** | **VigilMedian** | **PlaceboMedian** | **Difference** | **nVigil** | **nPlacebo** | **PVAL** |
| --- | --- | --- | --- | --- | --- | --- | --- |
| BRCA2wt | BRCA1m | 17.577 | 16 | 1.577 | 5 | 6 | 0.4216 |
| BRCA2wt | BRCA1wt | 18.3326 | 14.7844 | 3.5483 | 41 | 30 | 0.0274 |
| *BRCA2m-BRCA1m and BRCA2m-BRCA1wt were unable to be performed due to small sample size | | | | | | | |

| **Gene 1** | **Gene 2** | **VigilMedian** | **PlaceboMedian** | **Difference** | **nVigil** | **nPlacebo** | **PVAL** |
| --- | --- | --- | --- | --- | --- | --- | --- |
| ERCC2wt | BRCA1m | 17.577 | 16 | 1.577 | 5 | 7 | 0.4765 |
| ERCC2wt | BRCA1wt | 18.3326 | 14.9158 | 3.4168 | 42 | 34 | 0.0595 |
| * ERCC2m-BRCA1m and ERCC2m-BRCA1wt were unable to be performed due to small sample size | | | | | | | |

| **Gene 1** | **Gene 2** | **VigilMedian** | **PlaceboMedian** | **Difference** | **nVigil** | **nPlacebo** | **PVAL** |
| --- | --- | --- | --- | --- | --- | --- | --- |
| ERCC2wt | BRCA2wt | 18.2998 | 14.9158 | 3.384 | 46 | 34 | 0.0334 |
| *ERCC2m – BRCA2m, ERCC2m – BRCA2wt, ERCC2wt – BRCA2m were unable to be performed due to small sample size | | | | | | | |

| **Gene 1** | **Gene 2** | **VigilMedian** | **PlaceboMedian** | **Difference** | **nVigil** | **nPlacebo** | **PVAL** |
| --- | --- | --- | --- | --- | --- | --- | --- |
| HTR2Cm | BRCA2wt | 18.2998 | 17.7741 | 0.5257 | 17 | 5 | 0.2549 |
| HTR2Cwt | BRCA2wt | 19.6468 | 14.9158 | 4.731 | 29 | 31 | 0.0466 |
| *HTR2Cm-BRCA2m and HTR2Cwt-BRCA2m were unable to be performed due to small sample size | | | | | | | |

| **Gene 1** | **Gene 2** | **VigilMedian** | **PlaceboMedian** | **Difference** | **nVigil** | **nPlacebo** | **PVAL** |
| --- | --- | --- | --- | --- | --- | --- | --- |
| MUTYHwt | BRCA2wt | 18.3326 | 14.9158 | 3.4168 | 44 | 36 | 0.0302 |
| *MUTYHm – BRCA2m, MUTYHm – BRCA2wt, MUTYHwt – BRCA2m were unable to be performed due to small sample size | | | | | | | |

| **Gene 1** | **Gene 2** | **VigilMedian** | **PlaceboMedian** | **Difference** | **nVigil** | **nPlacebo** | **PVAL** |
| --- | --- | --- | --- | --- | --- | --- | --- |
| MYCNm | BRCA2wt | 17.347 | 15.9343 | 1.4127 | 13 | 12 | 0.185 |
| MYCNwt | BRCA2wt | 18.2998 | 14.7844 | 3.5154 | 33 | 24 | 0.0591 |
| *MYCNm – BRCA2m and MYCN– BRCA2m were unable to be performed due to small sample size | | | | | | | |

| **Gene 1** | **Gene 2** | **VigilMedian** | **PlaceboMedian** | **Difference** | **nVigil** | **nPlacebo** | **PVAL** |
| --- | --- | --- | --- | --- | --- | --- | --- |
| NF1wt | BRCA2wt | 18.2998 | 14.9158 | 3.384 | 46 | 35 | 0.0396 |
| * NF1m – BRCA2m, NF1m – BRCA2wt, NF1wt – BRCA2m were unable to be performed due to small sample size | | | | | | | |

| **Gene 1** | **Gene 2** | **VigilMedian** | **PlaceboMedian** | **Difference** | **nVigil** | **nPlacebo** | **PVAL** |
| --- | --- | --- | --- | --- | --- | --- | --- |
| OGG1wt | BRCA2wt | 18.2998 | 14.9158 | 3.384 | 45 | 35 | 0.033 |
| * OGG1m – BRCA2m, OGG1m – BRCA2wt, OGG1wt – BRCA2m were unable to be performed due to small sample size | | | | | | | |

| **Gene 1** | **Gene 2** | **VigilMedian** | **PlaceboMedian** | **Difference** | **nVigil** | **nPlacebo** | **PVAL** |
| --- | --- | --- | --- | --- | --- | --- | --- |
| PIK3CAwt | BRCA2wt | 18.2998 | 14.9158 | 3.384 | 45 | 35 | 0.0213 |
| * PIK3CAm – BRCA2m, PIK3CAm – BRCA2wt, PIK3CAwt – BRCA2m were unable to be performed due to small sample size | | | | | | | |

| **Gene 1** | **Gene 2** | **VigilMedian** | **PlaceboMedian** | **Difference** | **nVigil** | **nPlacebo** | **PVAL** |
| --- | --- | --- | --- | --- | --- | --- | --- |
| PRKNwt | BRCA2wt | 18.2998 | 14.9158 | 3.384 | 44 | 35 | 0.0227 |
| * PRKNm – BRCA2m, PRKNm – BRCA2wt, PRKNwt – BRCA2m were unable to be performed due to small sample size | | | | | | | |

| **Gene 1** | **Gene 2** | **VigilMedian** | **PlaceboMedian** | **Difference** | **nVigil** | **nPlacebo** | **PVAL** |
| --- | --- | --- | --- | --- | --- | --- | --- |
| RAD51Cwt | BRCA2wt | 18.3326 | 15.8686 | 2.4641 | 45 | 35 | 0.029 |
| * RAD51Cm – BRCA2m, RAD51Cm – BRCA2wt, RAD51Cwt – BRCA2m were unable to be performed due to small sample size | | | | | | | |

| **Gene 1** | **Gene 2** | **VigilMedian** | **PlaceboMedian** | **Difference** | **nVigil** | **nPlacebo** | **PVAL** |
| --- | --- | --- | --- | --- | --- | --- | --- |
| RUNX1wt | BRCA2wt | 18.3326 | 14.9158 | 3.4168 | 45 | 35 | 0.0241 |
| * RUNX1m – BRCA2m, RUNX1m – BRCA2wt, RUNX1wt – BRCA2m were unable to be performed due to small sample size | | | | | | | |

| **Gene 1** | **Gene 2** | **VigilMedian** | **PlaceboMedian** | **Difference** | **nVigil** | **nPlacebo** | **PVAL** |
| --- | --- | --- | --- | --- | --- | --- | --- |
| NF1wt | ERCC2wt | 18.3326 | 15.8686 | 2.4641 | 47 | 39 | 0.0516 |
| * NF1m – ERCC2m, NF1m – ERCC2wt, NF1wt – ERCC2m were unable to be performed due to small sample size | | | | | | | |

| **Gene 1** | **Gene 2** | **VigilMedian** | **PlaceboMedian** | **Difference** | **nVigil** | **nPlacebo** | **PVAL** |
| --- | --- | --- | --- | --- | --- | --- | --- |
| PIK3CAwt | ERCC2wt | 18.2998 | 15.8686 | 2.4312 | 46 | 40 | 0.0555 |
| * PIK3CAm – ERCC2m, PIK3CAm – ERCC2wt, PIK3CAwt – ERCC2m were unable to be performed due to small sample size | | | | | | | |

| **Gene 1** | **Gene 2** | **VigilMedian** | **PlaceboMedian** | **Difference** | **nVigil** | **nPlacebo** | **PVAL** |
| --- | --- | --- | --- | --- | --- | --- | --- |
| PRKNwt | ERCC2wt | 18.3326 | 15.8686 | 2.4641 | 45 | 40 | 0.0573 |
| * PRKNm – ERCC2m, PRKNm – ERCC2wt, PRKNwt – ERCC2m were unable to be performed due to small sample size | | | | | | | |

| **Gene 1** | **Gene 2** | **VigilMedian** | **PlaceboMedian** | **Difference** | **nVigil** | **nPlacebo** | **PVAL** |
| --- | --- | --- | --- | --- | --- | --- | --- |
| RUNX1wt | ERCC2wt | 18.3326 | 15.8686 | 2.4641 | 46 | 40 | 0.0536 |
| * RUNX1m – ERCC2m, RUNX1m – ERCC2wt, RUNX1wt – ERCC2m were unable to be performed due to small sample size | | | | | | | |

| **Gene 1** | **Gene 2** | **VigilMedian** | **PlaceboMedian** | **Difference** | **nVigil** | **nPlacebo** | **PVAL** |
| --- | --- | --- | --- | --- | --- | --- | --- |
| TP53m | ERCC2wt | 19.3511 | 15.8686 | 3.4825 | 33 | 30 | 0.0408 |
| TP53wt | ERCC2wt | 18.2998 | 25.5934 | -7.2936 | 14 | 11 | 0.4641 |
| * TP53m – ERCC2m, TP53wt– ERCC2m were unable to be performed due to small sample size | | | | | | | |

| **Gene 1** | **Gene 2** | **VigilMedian** | **PlaceboMedian** | **Difference** | **nVigil** | **nPlacebo** | **PVAL** |
| --- | --- | --- | --- | --- | --- | --- | --- |
| TP53m | MYCNm | NR | 15.9343 | NR | 7 | 8 | 0.0529 |
| TP53m | MYCNwt | 17.9713 | 17.7741 | 0.1971 | 26 | 24 | 0.2649 |
| TP53wt | MYCNm | 13.963 | 20.2218 | -6.2587 | 6 | 4 | 0.177 |
| TP53wt | MYCNwt | 19.6468 | 14.9158 | 4.731 | 8 | 8 | 0.195 |
| * NR indicates not reached | | | | | | | |

| **Gene 1** | **Gene 2** | **VigilMedian** | **PlaceboMedian** | **Difference** | **nVigil** | **nPlacebo** | **PVAL** |
| --- | --- | --- | --- | --- | --- | --- | --- |
| TP53m | NF1wt | 19.3511 | 16 | 3.3511 | 33 | 30 | 0.0577 |
| TP53wt | NF1wt | 18.2998 | 14.9158 | 3.384 | 14 | 12 | 0.4102 |
| * TP53m – NF1m, TP53wt– NF1m were unable to be performed due to small sample size | | | | | | | |
